# Site-specific and time-varying *Acinetobacter* colonization and risk of invasive infection in critically ill patients

**DOI:** 10.1007/s15010-026-02834-7

**Published:** 2026-05-29

**Authors:** Alessandro Russo, Sara Palma Gullì, Eugenio Garofalo, Vincenzo Olivadese, Helen Linda Morrone, Nadia Marascio, Giuseppe Neri, Andrea Bruni, Giovanni Matera, Angela Quirino, Francesca Serapide

**Affiliations:** 1 Infectious and Tropical Disease Unit, “Renato Dulbecco” University Hospital, Catanzaro, Italy; 2 Department of Medical and Surgical Sciences, “Magna Graecia” University, 88100 Catanzaro, Italy; 3 Intensive Care Unit, Department of Medical and Surgical Sciences, “Magna Graecia” University of Catanzaro, Catanzaro, Italy; 4 Department of Health Sciences, “Magna Graecia” University, Catanzaro, Italy; 5 Department of Pharmacy and Health and Nutritional Sciences, University of Calabria, Rende, Italy

**Keywords:** *Acinetobacter baumannii*, Colonization, Multisite colonization, Pneumonia, Bacteremia

## Abstract

**Purpose:**

Colonization by *Acinetobacter baumannii* is common among critically ill patients. However, the relative contribution of colonization at different anatomical sites and the impact of dynamic changes in colonization status over time remain incompletely defined.

**Methods:**

We conducted a retrospective cohort study of adult intensive care unit (ICU) patients with at least one surveillance culture positive for carbapenem-resistant *Acinetobacter* spp. (CRAB) between January 2022 and January 2026. Active surveillance cultures were obtained at ICU admission and weekly thereafter from respiratory, rectal, and skin sites. Progression to invasive CRAB infection (pneumonia and/or bloodstream infection) was assessed using multivariable logistic regression and Cox proportional hazards models with site-specific, time-updated colonization as exposures. Sensitivity analyses excluded infections occurring within 72 h of ICU admission.

**Results:**

Among 489 colonized ICU patients, 313 (64.0%) progressed to invasive CRAB infection during ICU stay. Respiratory colonization at ICU admission was strongly associated with progression to infection (adjusted OR, 5.46; 95% CI, 3.18–9.37), whereas rectal colonization was not independently associated with risk. In time-updated Cox models, respiratory (adjusted HR, 1.72; 95% CI, 1.35–2.18) and skin colonization (adjusted HR, 1.65; 95% CI, 1.31–2.08) were independently associated with infection risk. Colonization at multiple anatomical sites conferred additional risk.

**Conclusions:**

Progression to invasive infection was common and strongly associated with site-specific and time-varying colonization patterns, particularly involving the respiratory tract. Incorporating dynamic, multisite colonization data into risk assessment may improve identification of patients at highest risk for invasive infection and inform targeted infection prevention strategies in the ICU.

**Graphical abstract:**

**Figure:**
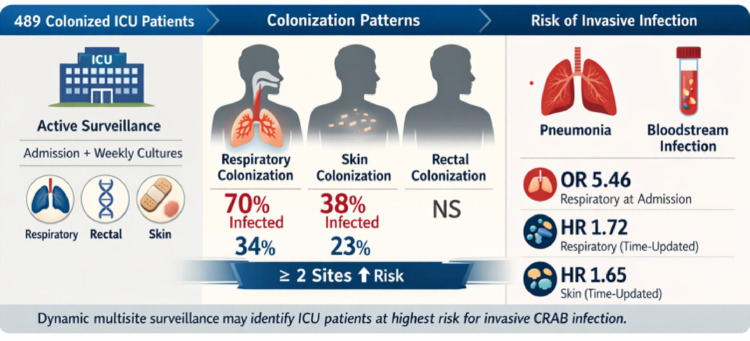

## Introduction

Carbapenem-resistant *Acinetobacter baumannii* (CRAB) has emerged as a major cause of healthcare-associated infections, particularly in intensive care unit (ICU) settings, where critically ill patients are exposed to prolonged hospitalization, invasive devices, and broad-spectrum antimicrobial therapy [1–4]. Invasive infections caused by CRAB are associated with limited therapeutic options and substantial morbidity and mortality, making prevention strategies a priority in contemporary critical care medicine [5, 6].

Colonization by CRAB is common among ICU patients and is considered a prerequisite for subsequent invasive infection. Active surveillance programs have demonstrated that colonization often precedes infection, particularly for ventilator-associated pneumonia (VAP) and bloodstream infection (BSI). However, not all colonized patients progress to invasive disease, and the determinants of this transition remain incompletely understood. In particular, the relative contribution of colonization at different anatomical sites and the dynamic nature of colonization during ICU stay have not been fully elucidated [6–9].

Previous studies have largely focused on point-prevalence colonization or have evaluated colonization at a single anatomical site, most commonly the respiratory tract [6–9]. Moreover, many analyses have treated colonization as a static exposure assessed at ICU admission, without accounting for changes over time. This approach may be insufficient in the ICU setting, where colonization status can evolve rapidly under the influence of antibiotic pressure, invasive procedures, and severity of illness. As a result, the true impact of site-specific and time-varying colonization on the risk of invasive CRAB infection remains uncertain.

In this study, we aimed to evaluate the risk of progression from colonization to invasive CRAB infection in a cohort of critically ill patients undergoing active surveillance cultures at ICU admission and during follow-up. Specifically, we assessed the association between colonization at different anatomical sites and the development of invasive infection, including the impact of illness severity, ICU-related exposures, and antibiotic pressure.

## Methods

### Study design and setting

We conducted a retrospective, observational cohort study including adult patients admitted to the ICU of 2 tertiary-care hospital who were colonized with CRAB during their ICU stay from January 2022 to January 2026. The study period and data collection were based on routinely collected clinical and microbiological data. The study was conducted in accordance with institutional regulations on observational research and was approved by local Ethical committee.

### Study population

All consecutive patients aged ≥ 18 years admitted to the ICU who had at least one positive surveillance culture for *Acinetobacter* spp. were eligible for inclusion. Patients were classified into two groups: those who remained colonized without developing invasive infection and those who progressed from colonization to invasive *Acinetobacter* infection during ICU stay. The identification of strains was performed by conventional and proteomic methods. Gram staining and a rapid identification protocol were adopted. The bacterial pellet obtained directly from positive blood cultures was used for MALDI-TOF MS (Bruker Daltonics) identification. The Vitek 2 automated system (bioMérieux, Florence, Italy) was used to identify bacterial strains and to test antimicrobial susceptibility following the manufacturer’s instructions. Patients with documented invasive CRAB infection at or before ICU admission were excluded.

### Active surveillance and colonization assessment

Active surveillance cultures were routinely obtained from all ICU patients at ICU admission and subsequently at weekly intervals. Surveillance samples included respiratory specimens (endotracheal aspirate or sputum), rectal swabs, and skin swabs. Skin surveillance cultures were routinely obtained from axillary and inguinal sites using standard screening procedures employed in participating ICUs. Colonization was defined as the isolation of carbapenem-resistant *Acinetobacter* spp. from surveillance cultures in the absence of clinical signs or symptoms of infection. Colonization status was recorded by anatomical site and treated as both a baseline (admission) and time-updated variable.

### Definitions of invasive infection

Invasive CRAB infection was defined as either pneumonia and/or bloodstream infection caused by *Acinetobacter* spp. Pneumonia was diagnosed based on the presence of new or progressive pulmonary infiltrates on chest imaging in association with compatible clinical signs and microbiological isolation of CRAB from respiratory samples. BSI was defined by at least one positive blood culture for CRAB with clinical signs of infection. Patients could experience more than one infection phenotype (pneumonia and bacteremia).

### Clinical variables and outcomes

Baseline demographic characteristics, comorbidities, and severity of illness at ICU admission were collected, including Acute Physiology and Chronic Health Evaluation II (APACHE II) and Sequential Organ Failure Assessment (SOFA) scores. ICU-related exposures included mechanical ventilation, duration of invasive ventilation, central venous catheter use, renal replacement therapy, and antibiotic exposure. Clinical outcomes included ICU length of stay, ICU mortality, and hospital mortality. Outcome variables were reported descriptively and were not included as covariates in multivariable models assessing risk factors for invasive infection.

### Antibiotic exposure

Antibiotic exposure was assessed longitudinally and treated as a time-dependent variable. Antibiotic use was summarized as patient-weeks of exposure and categorized by number of antibiotic classes and by specific antibiotic groups, including carbapenems, beta-lactam/beta-lactamase inhibitor combinations, cephalosporins, fluoroquinolones, aminoglycosides, colistin and other classes of antibiotics. Antibiotic escalation and de-escalation during ICU stay were also recorded. These analyses were descriptive and aimed to contextualize colonization dynamics.

### Statistical analysis

Continuous variables were summarized as mean ± standard deviation (SD) or median with interquartile range (IQR), as appropriate, and categorical variables as counts and percentages. Comparisons between patients who remained colonized and those who progressed to invasive infection were performed using the Student’s *t* test or the Mann–Whitney *U* test for continuous variables and the χ² test or Fisher’s exact test for categorical variables, as appropriate. P values from univariable comparisons were considered descriptive only.

To identify factors independently associated with progression from colonization to invasive infection, we fitted a multivariable logistic regression model. Covariates were selected a priori based on clinical relevance and included age, sex, comorbidities, APACHE II and SOFA scores at ICU admission, mechanical ventilation, central venous catheter use, and colonization site at ICU admission (respiratory, rectal, and skin). Results are reported as adjusted odds ratios (ORs) with 95% confidence intervals (CIs).

Time-to-event analyses were performed using Cox proportional hazards models to account for the dynamic nature of colonization. Time to invasive infection was calculated from ICU admission to the first episode of invasive infection, ICU discharge, or death, whichever occurred first. Colonization status by site was modeled as a time-updated covariate and updated weekly according to surveillance cultures. Models were adjusted for baseline severity of illness, immunosuppression, and major ICU exposures. Antibiotic exposure was considered a potential mediator in the pathway linking severity of illness, ICU-related exposures, and colonization dynamics with progression to invasive infection; therefore, it was not included in causal multivariable models to avoid overadjustment. Hazard ratios (HRs) with 95% CIs were reported.

Proportional hazards assumptions were assessed graphically using log-minus-log survival plots and Schoenfeld residual inspection and were deemed acceptable.

Kaplan–Meier curves were constructed to estimate infection-free survival and were stratified according to respiratory colonization status at ICU admission. The curves represent infection-free survival with censoring at: ICU discharge, death, or end of follow-up.

Sensitivity analyses were conducted to assess the robustness of the findings. Multivariable logistic regression analyses were repeated after excluding patients who developed invasive infection within 72 h of ICU admission, to reduce the risk of misclassification of infections present on admission and reverse causation. Results from sensitivity analyses were compared qualitatively with those from the primary analysis.

All statistical tests were two-sided, and a p value < 0.05 was considered statistically significant. Statistical analyses were performed using standard statistical software (IBM SPSS Statistics, version 28.0).

## Results

During the study period, 489 ICU patients with at least one surveillance culture positive for *Acinetobacter* spp. were included in the analysis. Among these, 176 patients (36.0%) remained colonized without developing invasive infection, whereas 313 patients (64.0%) progressed from colonization to invasive CRAB infection during ICU stay. Among infected patients, invasive infection manifested as pneumonia, BSI, or both.

Baseline characteristics and clinical outcomes of the study population are summarized in Table 1. Patients who progressed to invasive infection were older and had a higher burden of comorbidities, including chronic obstructive pulmonary disease and chronic kidney disease, compared with patients who remained colonized. Severity of illness at ICU admission was significantly higher among patients who developed infection, as reflected by higher APACHE II and SOFA scores and a higher prevalence of septic shock at admission. Exposure to invasive ICU procedures was more frequent among patients who developed invasive infection, including mechanical ventilation, prolonged invasive ventilation, central venous catheter use, and renal replacement therapy. Colonization patterns differed substantially between groups. Respiratory and skin colonization at ICU admission were significantly more frequent among patients who progressed to invasive infection, as was colonization at multiple anatomical sites. Rectal colonization at admission was numerically more frequent among infected patients but did not reach statistical significance. Patients who developed invasive infection had significantly longer ICU stays and higher ICU and hospital mortality compared with patients who remained colonized.

**Table 1:** Baseline characteristics and outcomes of ICU patients colonized with CRAB

Variable	Colonized only (*n* = 176)	Colonization → invasive infection (*n* = 313)	*p*-value
Age, years	61.8 ± 12.9	66.2 ± 11.2	**0.002**
Male sex	102 (58.0%)	206 (65.8%)	0.08
BMI, kg/m²	26.1 ± 4.8	27.4 ± 5.2	**0.01**
Comorbidities			
Diabetes mellitus	55 (31.2%)	112 (35.8%)	0.29
COPD	41 (23.3%)	107 (34.2%)	**0.01**
Chronic kidney disease	25 (14.2%)	88 (28.1%)	**< 0.001**
Chronic liver disease	12 (6.8%)	39 (12.5%)	**0.04**
Cardiovascular disease	69 (39.2%)	153 (48.9%)	**0.03**
Immunosuppression	26 (14.8%)	56 (17.9%)	0.37
Severity at ICU admission			
APACHE II score	18.4 ± 4.7	22.2 ± 5.8	**< 0.001**
SOFA score	6.5 ± 2.7	9.0 ± 3.0	**< 0.001**
Septic shock at admission	21 (11.9%)	74 (23.6%)	**0.002**
Mechanical ventilation	73 (41.5%)	235 (75.1%)	**< 0.001**
Invasive ventilation > 48 h	58 (33.0%)	211 (67.4%)	**< 0.001**
Central venous catheter	111 (63.1%)	258 (82.4%)	**< 0.001**
Renal replacement therapy	19 (10.8%)	61 (19.5%)	**0.01**
Colonization characteristics at ICU admission			
Respiratory colonization	60 (34.1%)	219 (70.0%)	**< 0.001**
Rectal colonization	79 (44.9%)	169 (54.0%)	0.06
Skin colonization	41 (23.3%)	119 (38.0%)	**0.001**
≥ 2 colonized sites	63 (35.8%)	187 (59.7%)	**< 0.001**
Outcome			
ICU length of stay, days	6.6 ± 4.4	12.9 ± 7.5	**< 0.001**
ICU mortality	18 (10.2%)	84 (26.8%)	**< 0.001**
In-hospital mortality	24 (13.6%)	111 (35.5%)	**< 0.001**

*p*-value that are statistically significant are in bold

*CRAB* Carbapenem-resistant *Acinetobacter baumannii,* *BMI* Body mass index, *COPD* Chronic obstructive pulmonary disease, *APACHE* Acute physiology chronic health evaluation, *SOFA* Sequential organ failure assessment, *ICU* Intensive care unit

**Fig. 1:**
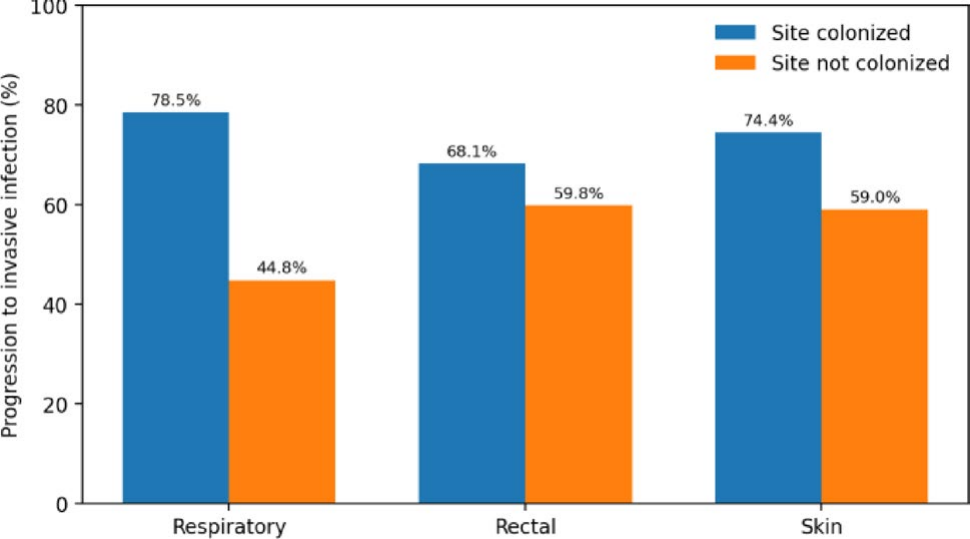
Invasive infections by colonization site

The proportion of patients developing invasive infection according to colonization status by anatomical site is shown in Fig. 1. Respiratory site was the most frequent colonized by CRAB, compared to rectal site.

**Fig. 2:**
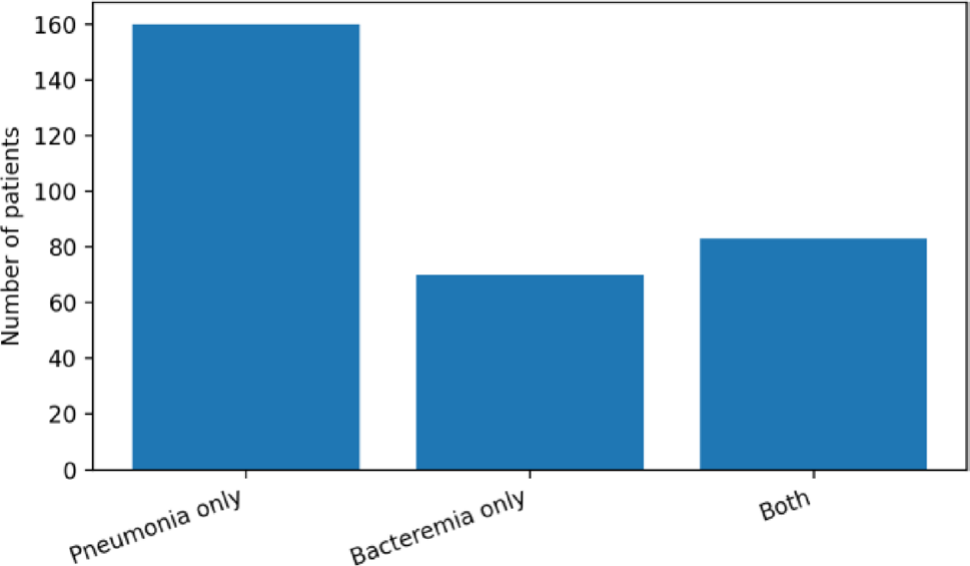
Site of CRAB infection. *CRAB* Carbapenem-resistant *Acinetobacter baumannii*

About infections, pneumonia (51.1%) was the most frequent infection followed by bacteremic pneumonia (26.5%), and primary bacteremia (22.4%), as reported in Fig. 2.

In multivariable logistic regression analysis adjusting for demographic characteristics, comorbidities, severity of illness, and ICU exposures, respiratory colonization at ICU admission remained independently associated with progression to invasive infection (adjusted OR 5.46, 95% CI 3.18–9.37; Table 2). Skin colonization and ≥ 2 colonized sites were also associated with increased risk, whereas rectal colonization was not independently associated with infection after adjustment.

**Table 2:** Multivariable logistic regression analysis for progression to invasive CRAB infection from colonization status

Variable	Adjusted OR	95% CI	*p*-value
Respiratory colonization at admission	5.46	3.18–9.37	< 0.001
Skin colonization at admission	1.77	0.99–3.15	0.05
Rectal colonization at admission	1.64	0.96–2.79	0.07
≥ 2 colonized sites	1.55	1.14–2.26	< 0.001

*CRAB* Carbapenem-resistant *Acinetobacter baumannii*

**Fig. 3:**
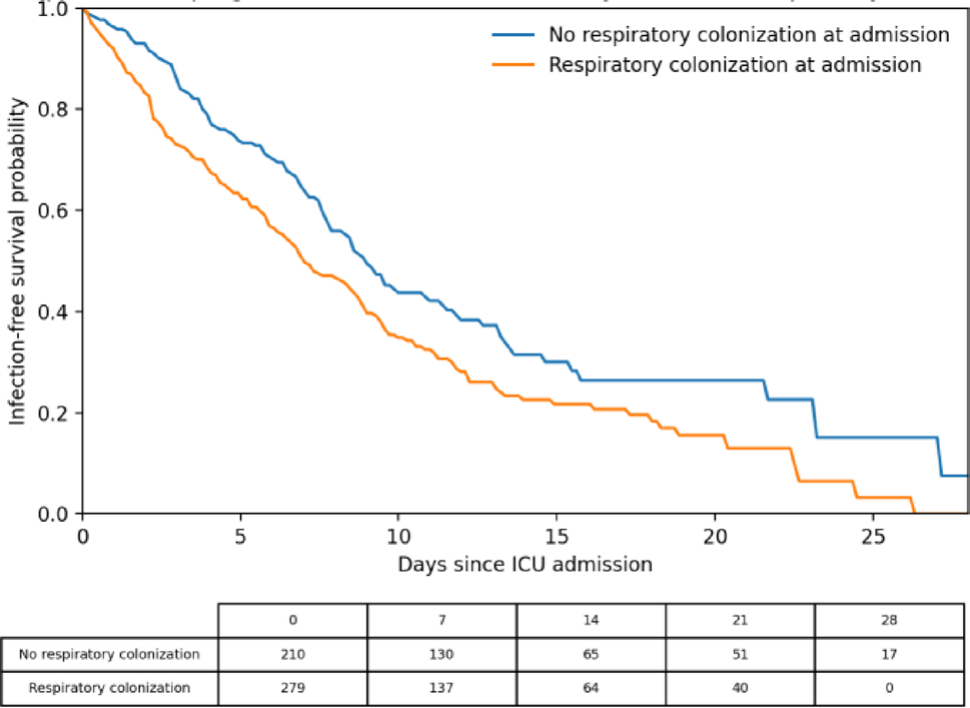
Kaplan-Meier curves about infection-free 30-day survival*. *p < 0.001

In Kaplan–Meier analysis, infection-free survival was significantly lower among patients with respiratory colonization at ICU admission compared with those without respiratory colonization (*p* < 0.001; Fig. 3).

To account for changes in colonization status over time, a Cox proportional hazards model with time-updated colonization variables was performed (Table 3). In this analysis, respiratory colonization treated as a time-updated exposure remained independently associated with progression to invasive infection (adjusted HR 1.72, 95% CI 1.35–2.18). Skin colonization was also independently associated with increased hazard of infection, whereas rectal colonization was not. Moreover, ≥ 2 colonized sites was independently associated with infection risk in the time-updated model.

**Table 3:** Time-updated Cox proportional hazards model for progression to invasive CRAB infection from colonization status*

Variable	Adjusted HR	95% CI	*p*-value
Respiratory colonization (time-updated)	1.72	1.35–2.18	< 0.001
Skin colonization (time-updated)	1.65	1.31–2.08	< 0.001
Rectal colonization (time-updated)	1.08	0.86–1.35	0.52
≥ 2 colonized sites	1.56	1.02–3.10	0.005

*CRAB* Carbapenem-resistant *Acinetobacter baumannii*

*Colonization status was updated weekly based on surveillance cultures

In sensitivity analyses excluding patients who developed invasive infection within 72 h of ICU admission, respiratory colonization at admission and ≥ 2 colonized sites remained strongly associated with progression to invasive infection, with effect estimates consistent with the primary analysis (Table 4).

**Table 4:** Sensitivity analysis excluding early infections (< 72 h) for progression to invasive CRAB infection)

Variable	Late-only analysis adj. OR (95% CI)	*p*-value
Age (per 1-year increase)	1.05 (1.02–1.07)	**< 0.001**
APACHE II (per point)	1.14 (1.08–1.19)	**< 0.001**
SOFA (per point)	1.47 (1.32–1.64)	**< 0.001**
Mechanical ventilation	6.06 (3.39–10.84)	**< 0.001**
≥ 2 colonized sites	2.55 (1.35–4.84)	**0.004**
Respiratory colonization at admission	5.18 (2.94–9.15)	**< 0.001**
Rectal colonization at admission	1.07 (0.61–1.86)	0.82
Skin colonization at admission	1.87 (1.00–3.51)	0.05

*p*-value that are statistically significant are in bold

*CRAB* Carbapenem-resistant *Acinetobacter baumannii,* *APACHE* Acute physiology chronic health evaluation, *SOFA* Sequential organ failure assessment

Moreover, antibiotic exposure during ICU stay was common and differed between groups (Table 5). Patients who progressed to invasive infection had a higher proportion of patient-weeks with antibiotic exposure, were more frequently exposed to multiple antibiotic classes, and more often received carbapenems and colistin. Prolonged antibiotic courses and escalation to broader-spectrum therapy were more common among patients who developed infection.

**Table 5:** Time-dependent antibiotic exposure during ICU stay (weekly-updated antibiotic exposure before progression to invasive infection)

Antibiotic exposure (time-updated)	Colonized only (*n* = 176)	Colonization → invasive infection (*n* = 313)	*p*-value
Any systemic antibiotic	412/702 patient-weeks (58.7%)	1,028/1,274 patient-weeks (80.7%)	< 0.001
Number of antibiotic classes			
0 classes	290 (41.3%)	246 (19.3%)	< 0.001
1 class	266 (37.9%)	403 (31.6%)	0.005
≥ 2 classes	146 (20.8%)	625 (49.1%)	< 0.001
Antibiotic classes			
Carbapenems	158 (22.5%)	566 (44.4%)	< 0.001
Piperacillin–tazobactam	133 (18.9%)	388 (30.4%)	< 0.001
Cephalosporins	119 (16.9%)	302 (23.7%)	< 0.001
Fluoroquinolones	84 (12.0%)	214 (16.8%)	0.004
Others (aminoglycosides, tetracycline, fosfomycin)	49 (7.0%)	192 (15.1%)	< 0.001
Colistin	21 (3.0%)	143 (11.2%)	< 0.001
Antibiotic pressure indicators			
≥ 7 consecutive days of antibiotics	197 (28.1%)	682 (53.5%)	< 0.001
Escalation to broader-spectrum therapy	64 (9.1%)	271 (21.3%)	< 0.001
De-escalation during ICU stay	118 (16.8%)	119 (9.3%)	< 0.001

*ICU* Intensive care unit

Finally, in Table 6 is reported risk of infection according to anatomical site and colonization status.

**Table 6:** Risk of infection according to anatomical site and colonization status

Colonization variable	Colonized infected	Colonized not infected	Colonized total	Risk if colonized (%)	Not colonized infected	Not colonized not infected	Not colonized total	Risk if not colonized (%)	Crude OR	95% CI	*p*-value
Respiratory colonization at ICU admission	219	60	279	78.5	94	116	210	44.8	4.50	3.04–6.68	< 0.001
Rectal colonization at ICU admission	169	79	248	68.1	144	97	241	59.8	1.44	0.99–2.09	0.053
Skin colonization at ICU admission	119	41	160	74.4	194	135	329	59.0	2.02	1.33–3.07	< 0.001
≥ 2 colonized sites at ICU admission	187	63	250	74.8	126	113	239	52.7	2.66	1.82–3.90	< 0.001

*ICU* Intensive care unit

## Discussion

In this cohort of critically ill patients undergoing active surveillance for *Acinetobacter* colonization, we found that progression from colonization to invasive infection was frequent and strongly associated with both site-specific and dynamic colonization patterns. In particular, respiratory colonization at ICU admission and during follow-up emerged as the strongest and most consistent predictor of invasive infection, while skin colonization also conferred an increased risk. These associations remained robust after adjustment for severity of illness, ICU-related exposures, and in sensitivity analyses excluding early infections.

Our findings confirm and extend previous observations that colonization precedes invasive *Acinetobacter* infection in the ICU setting [6]. While earlier studies have demonstrated an association between colonization and infection, most have relied on single-site or admission-only surveillance. By incorporating systematic screening at multiple anatomical sites and modeling colonization as a time-updated exposure, our analysis provides a more granular assessment of how colonization dynamics influence infection risk over time. Understanding which colonized patients are at highest risk of progression to invasive infection is clinically relevant for several reasons. First, it may inform targeted infection prevention strategies, including intensified surveillance or isolation measures. Second, it may help identify patients who could benefit from early diagnostic or therapeutic interventions. Finally, distinguishing colonization patterns that confer higher risk may improve the interpretation of surveillance cultures and guide antimicrobial stewardship efforts [12–14].

Respiratory tract colonization was consistently associated with the highest risk of subsequent invasive infection across all analytical approaches, including logistic regression, Kaplan–Meier analysis, and time-updated Cox modeling. This finding is biologically plausible, given the central role of the respiratory tract as both a reservoir and portal of entry for *Acinetobacter* in mechanically ventilated patients. The strong association observed even after excluding early infections suggests that respiratory colonization is not merely a marker of infection present at admission, but a meaningful predictor of future invasive disease. Although distinguishing respiratory colonization from early infection in mechanically ventilated patients remains challenging, the consistency of results across baseline, time-updated, and sensitivity analyses excluding early infections supports the robustness of this association [6–17].

Skin colonization was also independently associated with progression to invasive infection, particularly in baseline analyses. This observation highlights the potential role of skin as a reservoir for cross-transmission and device-related infections, especially in patients with multiple invasive lines. In contrast, rectal colonization alone was not independently associated with infection risk, suggesting that gastrointestinal carriage may be less relevant for invasive *Acinetobacter* disease than for other multidrug-resistant organisms.

Finally, multisite colonization showed an important role in development of invasive infection. This is in line with recent observations about the role of multiple sites colonization, suggesting the potential role in lower respiratory tract infections and the role of surveillance and infection control in critically ill patients. Multisite colonization may reflect a higher burden of bacterial exposure and impaired host defenses, thereby identifying a subgroup of patients at particularly high risk for invasive disease [6–20].

Severity of illness, as reflected by APACHE II and SOFA scores, were consistently associated with infection risk, underscoring the complex interplay between host vulnerability, ICU care, and microbial factors. Importantly, the association between colonization and infection persisted after adjustment for these variables, supporting the hypothesis that colonization contributes independently to infection risk rather than acting solely as a surrogate marker of severity [19, 20].

Antibiotic exposure was common in this cohort and was more frequent and prolonged among patients who developed invasive infection. Although antibiotic use was not modeled as a causal exposure, descriptive analyses suggest that antibiotic pressure may influence colonization dynamics and contribute to the selection and persistence of *Acinetobacter*. These findings emphasize the importance of antimicrobial stewardship in conjunction with surveillance-based infection prevention strategies [8, 21, 22].

Our sensitivity analysis excluding infections occurring within 72 h of ICU admission yielded results consistent with the primary analysis, strengthening the robustness of our findings and mitigating concerns regarding misclassification of infections present at admission or reverse causation. Together with the time-updated modeling approach, these analyses support a temporal relationship between colonization and subsequent invasive infection.

This study has several limitations. First, its observational design precludes causal inference, and residual confounding cannot be excluded. Second, data were derived from a cohort in a region with high endemicity of carbapenem-resistant *Acinetobacter*, which may limit generalizability to settings with different epidemiology or infection control practices [23]. Third, molecular typing was not performed, and therefore clonality between colonizing and infecting isolates could not be confirmed; it is important to consider also that pneumonia represented the predominant invasive infection phenotype. Moreover, antibiotic exposure was described but not incorporated as a time-updated covariate in causal models, and its role should be further explored in future studies. Finally, the absence of a non-colonized comparator group precluded assessment of the absolute risk of invasive infection associated with colonization compared with patients without detectable CRAB carriage.

Despite these limitations, our study has important clinical implications. The identification of respiratory and skin colonization as key risk factors for invasive *Acinetobacter* infection supports the use of targeted surveillance and reinforces the need for stringent infection prevention measures in colonized patients. Moreover, the dynamic nature of colonization observed in this cohort suggests that repeated surveillance may provide clinically relevant information beyond admission screening alone. The high rate of progression observed in this cohort should be interpreted in light of the study population, which included only colonized ICU patients in a setting with endemic carbapenem-resistant *Acinetobacter* and systematic active surveillance.

In conclusion, among critically ill patients colonized with *Acinetobacter*, progression to invasive infection is common and strongly associated with site-specific and time-varying colonization, particularly of the respiratory tract. Incorporating dynamic colonization data into risk assessment may improve identification of high-risk patients and inform targeted preventive strategies in the ICU.

## Data Availability

Data are available on request at a.russo@unicz.it.
